# Correction: Validity and reliability International Classification of Diseases-10 codes for all forms of injury: A systematic review

**DOI:** 10.1371/journal.pone.0318388

**Published:** 2025-01-24

**Authors:** The PLOS One Staff

## Notice of republication

This article was republished on January 16, 2025, to correct an error in the author list. Michael D. Cusimano was mistakenly omitted from the author byline. The publisher apologizes for this error. Please download the article again to view the corrected version. For reference, both the originally published, uncorrected article and the republished, corrected article are provided here.

## Supporting information

S1 File Originally published, uncorrected article. (PDF)

S2 File Republished, corrected article. (PDF)
