# Supplementary material for: Validity and reliability International Classification of Diseases-10 codes for all forms of injury: A systematic review
Source: PLoS One. 2024 Feb 29;19(2):e0298411. doi: 10.1371/journal.pone.0298411 (PMC10903801; doi:10.1371/journal.pone.0298411)
Supplement: S1 Text — (DOCX) [file pone.0298411.s002.docx]

**S1 Text. Ovid Medline Search**

**Ovid MEDLINE (April 16/2023):**

Epub Ahead of Print, In-Process & Other Non-Indexed Citations, Ovid MEDLINE® Daily and Ovid MEDLINE® <1946-Present>

1 Injur*.tw,kf. 1002545

2 Traumatic brain injur*.tw,kf. 47934

3 Transport incident*.tw,kf. 23

4 Crash*.tw,kf. 17122

5 Fall*.tw,kf. 247104

6 Drown*.tw,kf. 6167

7 Burn*.tw,kf. 122628

8 (Fire* adj3 injur*).tw,kf. 2608

9 Poisoning*.tw,kf. 80342

10 Violence.tw,kf. 63553

11 exp Accidents, Traffic/ or exp Accidents, Home/ or exp Accidents/ or exp Accidents, Occupational/ 208365

12 exp "Wounds and Injuries"/ 1002562

13 exp Accidental Falls/ 27923

14 exp Domestic Violence/ or exp Intimate Partner Violence/ 54597

15 exp Spouse Abuse/ or exp Physical Abuse/ or exp Child Abuse/ 41837

16 exp Fractures, Bone/ 206775

17 exp Hip Fractures/ 28497

18 exp Spinal Fractures/ 17487

19 1 or 2 or 3 or 4 or 5 or 6 or 7 or 8 or 9 or 10 or 11 or 12 or 13 or 14 or 15 or 16 or 17 or 18 2201294

20 Reliability.tw,kf. 208188

21 Validity.tw,kf. 212478

22 Validation.tw,kf. 296078

23 exp "Reproducibility of Results"/ 463148

24 exp "Sensitivity and Specificity"/ 645717

25 Sensitivity.tw,kf. 967906

26 Specificity.tw,kf. 550010

27 20 or 21 or 22 or 23 or 24 or 25 or 26 2325303

28 ICD 10*.tw,kf. 15328

29 ("International Classification of Diseases" and (tenth revision* or "10")).tw,kf. 6656

30 28 or 29 18903

31 19 and 27 and 30 313
