# Supplementary material for: Validity and reliability International Classification of Diseases-10 codes for all forms of injury: A systematic review
Source: PLoS One. 2024 Feb 29;19(2):e0298411. doi: 10.1371/journal.pone.0298411 (PMC10903801; doi:10.1371/journal.pone.0298411)
Supplement: S2 Text — (DOCX) [file pone.0298411.s003.docx]

**S2 Text. Cochrane Library Search**

**Cochrane Library (April 18/2023):**

ID Search Hits

#1 (Injur*):ti,ab,kw 74035

#2 (Traumatic brain injur*):ti,ab,kw 4868

#3 (Transport incident*):ti,ab,kw 66

#4 (Crash*):ti,ab,kw 755

#5 (Fall*):ti,ab,kw 27261

#6 (Drown*):ti,ab,kw 144

#7 (Burn*):ti,ab,kw 12640

#8 (Fire* near/3 injur*):ti,ab,kw 79

#9 (Poisoning*):ti,ab,kw 2382

#10 (Violence):ti,ab,kw 3907

#11 [mh "Accidents"] 21062

#12 [mh "Accidents, Traffic"] 543

#13 [mh "Accidents, Home"] 96

#14 [mh "Accidents, Occupational"] 112

#15 [mh "Wounds"] 34224

#16 [mh "Injuries"] 34224

#17 [mh "Accidental Falls"] 1921

#18 [mh "Domestic Violence"] 1121

#19 [mh "Intimate Partner Violence"] 553

#20 [mh "Spouse Abuse"] 224

#21 [mh "Physical Abuse"] 56

#22 [mh "Child Abuse"] 705

#23 [mh "Fractures, Bone"] 8166

#24 [mh "Hip Fractures"] 2154

#25 [mh "Spinal Fractures"] 973

#26 {or #1-#25} 144626

#27 (Reliability*):ti,ab,kw 13134

#28 (Validity*):ti,ab,kw 13168

#29 (Validation*):ti,ab,kw 16009

#30 [mh "Reproducibility of Results"] 14526

#31 [mh "Sensitivity and Specificity"] 19899

#32 (Sensitivity):ti,ab,kw 67965

#33 (Specificity):ti,ab,kw 23272

#34 {or #27-#33} 114285

#35 (ICD 10*):ti,ab,kw 3789

#36 ((International Classification of Diseases) and (tenth revision* or 10)):ti,ab,kw 1027

#37 {or #35-#36} 4432

#38 #26 and #34 and #37 38
