# Supplementary material for: Validity and reliability International Classification of Diseases-10 codes for all forms of injury: A systematic review
Source: PLoS One. 2024 Feb 29;19(2):e0298411. doi: 10.1371/journal.pone.0298411 (PMC10903801; doi:10.1371/journal.pone.0298411)
Supplement: S3 Text — (DOCX) [file pone.0298411.s004.docx]

**S3 Text. EMBASE Search**

**EMBASE (April 18/2023):**

Classic+Embase <1947 to 2023 April 17>

1 Injur*.tw,kf. 1358915

2 Traumatic brain injur*.tw,kf. 71747

3 Transport incident*.tw,kf. 30

4 Crash*.tw,kf. 20062

5 Fall*.tw,kf. 360147

6 Drown*.tw,kf. 8148

7 Burn*.tw,kf. 170456

8 (Fire* adj3 injur*).tw,kf. 3448

9 Poisoning*.tw,kf. 102171

10 Violence.tw,kf. 76517

11 exp Accidents, Traffic/ or exp Accidents, Home/ or exp Accidents/ or exp Accidents, Occupational/ 253050

12 exp "Wounds and Injuries"/ 2882368

13 exp Accidental Falls/ 48996

14 exp Domestic Violence/ or exp Intimate Partner Violence/ 73728

15 exp Spouse Abuse/ or exp Physical Abuse/ or exp Child Abuse/ 64374

16 exp Fractures, Bone/ 391898

17 exp Hip Fractures/ 49561

18 exp Spinal Fractures/ 38043

19 1 or 2 or 3 or 4 or 5 or 6 or 7 or 8 or 9 or 10 or 11 or 12 or 13 or 14 or 15 or 16 or 17 or 18 4022237

20 Reliability.tw,kf. 262617

21 Validity.tw,kf. 267250

22 Validation.tw,kf. 432243

23 exp "Reproducibility of Results"/ 257268

24 exp "Sensitivity and Specificity"/ 479426

25 Sensitivity.tw,kf. 1319191

26 Specificity.tw,kf. 747488

27 20 or 21 or 22 or 23 or 24 or 25 or 26 2654161

28 ICD 10*.tw,kf. 33394

29 ("International Classification of Diseases" and (tenth revision* or "10")).tw,kf. 9731

30 28 or 29 38613

31 19 and 27 and 30 484

32 Injur*.tw,kf. 1358915

33 Traumatic brain injur*.tw,kf. 71747

34 Transport incident*.tw,kf. 30

35 Crash*.tw,kf. 20062

36 Fall*.tw,kf. 360147

37 Drown*.tw,kf. 8148

38 Burn*.tw,kf. 170456

39 (Fire* adj3 injur*).tw,kf. 3448

40 Poisoning*.tw,kf. 102171

41 Violence.tw,kf. 76517

42 exp Accidents, Traffic/ or exp Accidents, Home/ or exp Accidents/ or exp Accidents, Occupational/ 253050

43 exp "Wounds and Injuries"/ 2882368

44 exp Accidental Falls/ 48996

45 exp Domestic Violence/ or exp Intimate Partner Violence/ 73728

46 exp Spouse Abuse/ or exp Physical Abuse/ or exp Child Abuse/ 64374

47 exp Fractures, Bone/ 391898

48 exp Hip Fractures/ 49561

49 exp Spinal Fractures/ 38043

50 32 or 33 or 34 or 35 or 36 or 37 or 38 or 39 or 40 or 41 or 42 or 43 or 44 or 45 or 46 or 47 or 48 or 49 4022237

51 Reliability.tw,kf. 262617

52 Validity.tw,kf. 267250

53 Validation.tw,kf. 432243

54 exp "Reproducibility of Results"/ 257268

55 exp "Sensitivity and Specificity"/ 479426

56 Sensitivity.tw,kf. 1319191

57 Specificity.tw,kf. 747488

58 51 or 52 or 53 or 54 or 55 or 56 or 57 2654161

59 ICD 10*.tw,kf. 33394

60 ("International Classification of Diseases" and (tenth revision* or "10")).tw,kf. 9731

61 59 or 60 38613

62 50 and 58 and 61 484
