# Supplementary material for: Validity and reliability International Classification of Diseases-10 codes for all forms of injury: A systematic review
Source: PLoS One. 2024 Feb 29;19(2):e0298411. doi: 10.1371/journal.pone.0298411 (PMC10903801; doi:10.1371/journal.pone.0298411)
Supplement: S4 Text — (DOCX) [file pone.0298411.s005.docx]

**S4 Text. Scopus Search**

**Scopus (April 19/2023):**

Advanced search

( TITLE-ABS-KEY ( injur*  OR  ( traumatic  AND  brain  AND  injur* )  OR  ( transport  AND  incident* )  OR  crash*  OR  fall*  OR  drown*  OR  burn*  OR  fire*  OR  poisoning*  OR  violence  OR  violent  OR  accident  OR  traffic  OR  wound*  OR  fracture* )  AND  TITLE-ABS-KEY ( ( ( reliability*  OR  validity*  OR  sensitivity  OR  specificity  OR  reliability*  OR  validation )  OR  ( reproducibility  AND  of  AND  results ) ) )  AND  TITLE ( ( ( icd  AND  10* )  OR  ( international  AND  classification  AND  of  AND  diseases  AND  tenth  AND  revision* )  OR  ( international  AND  classification  AND  of  AND  diseases  AND  10 ) ) )  AND  ABS ( ( ( icd  AND  10* )  OR  ( international  AND  classification  AND  of  AND  diseases  AND  tenth  AND  revision* )  OR  ( international  AND  classification  AND  of  AND  diseases  AND  10 ) ) ) )
