# Supplementary material for: Validity and reliability International Classification of Diseases-10 codes for all forms of injury: A systematic review
Source: PLoS One. 2024 Feb 29;19(2):e0298411. doi: 10.1371/journal.pone.0298411 (PMC10903801; doi:10.1371/journal.pone.0298411)

| Please consider “the test” as the diagnostic algorithm (i.e., assigning of ICD10 codes for reporting injuries – which we are reviewing the accuracy of) used in the paper. | | | | | |
| --- | --- | --- | --- | --- | --- |
| **The QUADAS tool** | | | | | |
| **Item** |  | **Yes (1)** | **No (0)** | **Unclear (0)** | **N/A (0)** |
| ------------------------------------------------------------------------------------------------------------------------------------------------------ | | | | | |
| 1. | Was the spectrum of patients representative of the patients who will receive the comorbidity diagnosis/test in practice (i.e., hospital/ED patients)? | ( ) | ( ) | ( ) | ( ) |
| 2. | Were selection criteria clearly described? | ( ) | ( ) | ( ) | ( ) |
| 3. | Is the reference standard likely to correctly classify the target condition (i.e., injury patients)? | ( ) | ( ) | ( ) | ( ) |
| 4. | ~~Is the time period between reference standard and index test short enough to be reasonably sure that the target condition did not change between the two tests?~~ (NA) | ( ) | ( ) | ( ) | ( ) |
| 5. | Did the whole sample or a random selection of the sample, receive verification using a reference standard of diagnosis? | ( ) | ( ) | ( ) | ( ) |
| 6. | Did patients receive the same reference standard regardless of the index test result? | ( ) | ( ) | ( ) | ( ) |
| 7. | Was the reference standard independent of the index test (i.e. the index test did not form part of the reference standard)? | ( ) | ( ) | ( ) | ( ) |
| 8. | Was the execution of the index test described in sufficient detail to permit replication of the test? | ( ) | ( ) | ( ) | ( ) |
| 9. | Was the execution of the reference standard described in sufficient detail to permit its replication? (i.e., chart review/physician diagnosis details included or not) | ( ) | ( ) | ( ) | ( ) |
| 10. | Were the index test results interpreted without knowledge of the results of the reference standard? | ( ) | ( ) | ( ) | ( ) |
| 11. | Were the reference standard results interpreted without knowledge of the results of the index test (i.e., without knowledge of what the chart/physician diagnosis reported)? | ( ) | ( ) | ( ) | ( ) |
| 12. | Were the same clinical data (i.e., chart review/physician diagnosis) available when test results were interpreted as would be available when the test is used in practice? | ( ) | ( ) | ( ) | ( ) |
| 13. | Were uninterpretable/ intermediate test results reported? | ( ) | ( ) | ( ) | ( ) |
| 14. | Were withdrawals from the study explained? | ( ) | ( ) | ( ) | ( ) |
|  | | | | | |
|  | **Total Score**  **Sum of applicable items: ___/13____ Number of N/A items: ______** | | | | |
|  | **Please select your opinion of the overall quality of this study** | Poor Excellent  (1) (2) (3) (4) (5) | | | |
| Comments  Note: | | | | | |


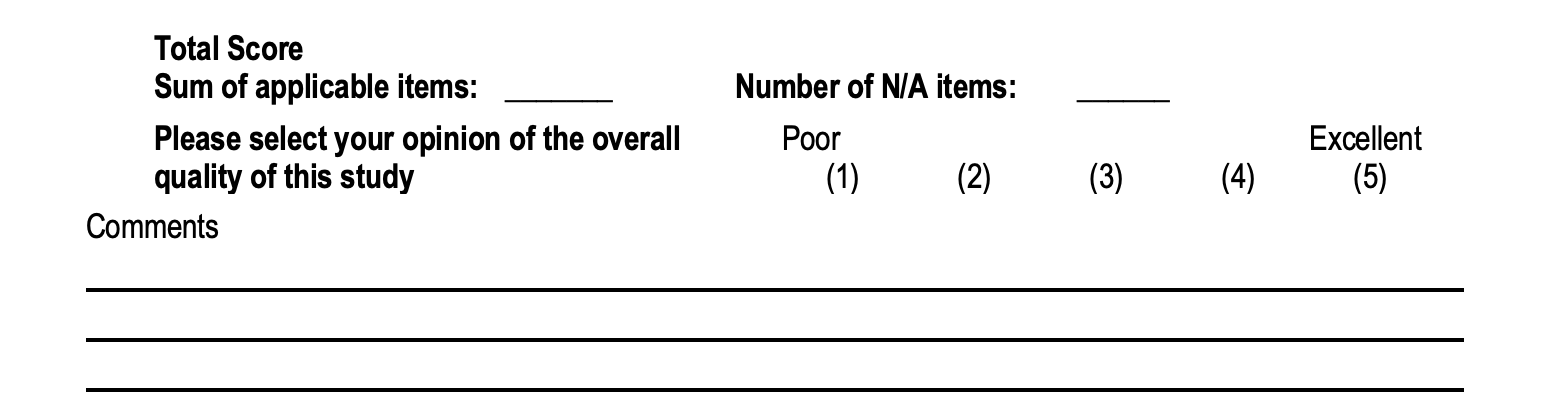

Supplement: S5 Text — (DOCX) [file pone.0298411.s006.docx]
