# Supplementary material for: Validity and reliability International Classification of Diseases-10 codes for all forms of injury: A systematic review
Source: PLoS One. 2024 Feb 29;19(2):e0298411. doi: 10.1371/journal.pone.0298411 (PMC10903801; doi:10.1371/journal.pone.0298411)
Supplement: S1 Table — (DOCX) [file pone.0298411.s007.docx]

**Table S1. Outcomes of all studies examined**

| Study | Injury codes description | Specific comparator identified within the injury codes analyzed* | Injury codes analyzed | Sample Size (n) | PPV, % (CI) | NPV, % (CI) | Sensitivity, % (CI) | Specificity, % (CI) | Inter-rater reliability | | | Comment(s) |
| --- | --- | --- | --- | --- | --- | --- | --- | --- | --- | --- | --- | --- |
|  |  |  |  |  |  |  |  |  | Krippendorff's alpha | Cohen’s Kappa | Fleiss’ kappa |  |
| **Transport and Pedestrian Injuries** | | | | | | | | | | | | |
| **(Karkhaneh et al., 2012)** | Bicycle injuries | Emergency department (ED) coder (2004) | Unspecified bicycle and pedestrian physical injuries ICD10 codes | n=120 | - | - | 96.7 (88.5-99.6) | - | - | 0.93 (0.87-0.99) | - | Independent refers to the independent coders (employed to assign both ICD-9-CM and ICD-10-CA codes for cases), in contrast to the emergency department (ED) coders (medical record nosologists that assigned ICD-9-CM or ICD-10-CA at the time of ED discharge) |
|  |  | ED coder (2007) |  |  | - | - | 90.2 (79.8- 96.3) | - | - | 0.88 (0.80-0.97) | - |  |
|  |  | Independent (IND) coder (2001) |  |  | - | - | 98.3 (91.1-100) | - | - | - | - |  |
|  |  | IND coder (2004) |  |  | - | - | 98.3 (91.1-100) | - | - | - | - |  |
|  |  | IND coder (2007) |  |  | - | - | 95.1 (86.3-99.0) | - | - | - | - |  |
|  | Pedestrian injuries | ED coder (2004) |  |  | - | - | 25.0 (14.7-37.9) | 100.0 | - | 0.90 (0.78-1.00) | - |  |
|  |  | ED coder (2007) |  |  | - | - | 35.6 (23.6-49.1) | 100.0 | - | 0.97 (0.92-1.00) | - |  |
|  |  | IND coder (2001) |  |  | - | - | 45.0 (32.1-58.4) | 100.0 | - | - | - |  |
|  |  | IND coder (2004) |  |  | - | - | 30.0 (18.8-43.2) | 100.0 | - | - | - |  |
|  |  | IND coder (2007) |  |  | - | - | 30.0 (18.8-43.2) | 100.0 | - | - | - |  |
| **(Henderson et al., 2006)** | Femur fractures; transport incident injuries; accidental poisoning and exposure to noxious substances | Motor vehicle accidents: 1998-1999 | V40-V59 | This study did not specify the portion of the sample size that fell under this comparator group, but the total sample size was n<14635 (an unspecified number were applicable for this study’s analysis) | 95 (89-100) | - | 87 (77-97) | - | - | 0.91 | - | Study outcomes split into outcomes for lower extremities injuries, transport and pedestrian injuries, and poisoning |
|  |  | Motor vehicle accidents: 2000-2001 |  |  | 98 (95-100) | - | 98 (95-100) | - | - | 0.98 | - |  |
| **Self-Harm Injuries** | | | | | | | | | | | | |
| **(Sveticic et al., 2020)** | Intentional self-harm | Identifying suicide attempt cases | X84 | n=130 | 56.3 (50.4-62.0) | 73.7 (73.0-74.4) | 18.7 (16.0-21.7) | 94.0 (92.8-95.1) | - | - | - |  |
|  |  | Identifying non-suicidal self-injury cases |  |  | 29.1 (24.6-34.1) | 95.0 (94.4-95.5) | 38.5 (31.5-45.9) | 92.6 (91.4-93.6) | - | - | - |  |
| **(Randall et al., 2017)** | Poisoning, intentional self-harm, and related events of undetermined intent | Suicide attempt patients identified using X60-X84, Y10-Y34, X40-X49; Self-harm patients identified using X60-X84, Y10-Y34, X40-X49 | X60-X84, Y10-Y34, X40-X49 | X60-X84, Y10-Y34, and X40-X49 (n=5719) | 66.8 (61.4-72.1); 83.6 (79.3-87.8) | 91.9 (90.9-92.9); 87.7 (86.5-88.9) | 44.8 (40.2-49.4); 40.0 (36.2-43.9) | 96.6 (95.9-97.2); 98.2 (97.7-98.7) | - | 0.481 (0.434–0.528); 0.478 (0.435–0.521) | - |  |
| **(Gabella et al., 2022)** | Suicide attempt and self-harm of multiple types | Based on physician diagnosis at hospital site 1^c^; hospital site 2^d^; hospital site 3^e^ | X71–X83, T14.91, T36–T50^f^, T51–T65^g^, T71^h^ | X71–X83 (n=414), T14.91 (n=36), T36–T50 (n=387), T51–T65 (n=46), T71 (n=28) *  *The codes that were analyzed in this study were not organized under the specific comparators identified. | 89.8; 91.9; 97.3 | - | - | - | - | - | - | ^c^Maryland  ^d^Colarado  ^e^Massachusetts  ^f^With the 6th character of the ICD10-CM code of “2,” except for T36.9, T37.9, T39.9, T41.4, T42.7, T43.9, T45.9, T47.9, and T49.9, which are included if the 5th character of the code is “2”  ^g^With the 6th character of the ICD-10-CM code of “2,” except for T51.9, T52.9, T53.9, T54.9, T56.9, T57.9, T58.0, T58.1, T58.9, T59.9, T60.9, T61.0, T61.1, T61.9, T62.9, T63.9, T64.0, T64.8, and T65.9, which are included if the 5th character of the code is “2”  ^h^With the 6th character of the ICD-10-CM code of “2” |
|  |  | Based on physician documentation or diagnosis at hospital site 1^c^; hospital site 2^d^; hospital site 3^e^ |  |  | 97.9;  98.4;  98.9 | - | - | - | - | - | - |  |
|  |  | Based on any documentation diagnosis at hospital site 1^c^; hospital site 2^d^; hospital site 3^e^ |  |  | 97.9;  98.8;  98.9 | - | - | - | - | - | - |  |
| **(Hansen et al., 2021)** | Suicide attempt; poisoning by drugs, medicaments, and biological substances; asphyxiation; intentional self-harm (multiple causes) | Intentional self-harm | T14.91, T36-T50, T54, T71, X78-X79, X80, X83 | T14.91 (n=7), T36-T50 (n=100), T54 (n<5), T71(n<5), X78 (n=82), X79 (n<5), X80 (n<5), X83 (n=10) | 88.9 (83.8-92.8) | - | - | - | - | - | - |  |
|  |  | Intentional self-harm with intent to die (i.e., suicide attempt) |  |  | 38.2 (31.5-45.2) | - | - | - | - | - | - |  |
|  |  | Suicidal ideation |  |  | 51.2 (44.2-58.2) | - | - | - | - | - | - |  |
| **Spinal Cord Injuries** | | | | | | | | | | | | |
| **(Furlan & Fehlings, 2011)** | Spinal injury outcomes (fractures, nerve injuries, lesions, and epidural haemorrhage) | Patients with spinal trauma | S06.400, S12.1, S12.900, S14.10, S22.000, S23.1, S24.10, S32.000, S32.100, T06.1 | S06.400 (n=2), S12.900 (n=12), S14.10 (n=3), S22.000 (n=14), S24.10 (n=1), S32.000 (n=53), S32.100 (n=4), T06.1 (n=1), S23.1 (n=1), S12.1 (n=1) | 96.3 (89-99) | 10 (0-44) | 89.8 (81-95) | 25 (0-80) | - | - | - |  |
|  |  | Patients with complete motor injury |  |  | 30 (20-41) | 40 (12-73) | 80 (61-92) | 6.7 (1-16) | - | - | - |  |
| **(Welk et al., 2014)** | Injuries of brain and spinal cord outcomes (including concussion, oedema, and nerve injuries) | Specification of lesion level:  Cervical | S14.0/S14.1 | n=114 | 97 (91-99) | 75 (67-82) | 75 (67-82) | 97 (91-99) | - | 0.70 | - | ^k^Concerning whether any of the codes were present within 60 or 180 days of the SCI date as determined from the chart abstraction data. |
|  |  | Specification of lesion level: Thoracic | S24.0/S24.1 | n=60 | 93 (81-98) | 86 (81-98) | 69 (51-75) | 98 (94-99) | - | 0.68 | - |  |
|  |  | Specification of lesion level: Lumbar | S34.0/S34.1/S34.3 | n=26 | 76 (53-90) | 93 (88-96) | 50 (32-68) | 98 (95-99) | - | 0.56 | - |  |
| **(Hagen et al., 2009)** | Acute traumatic spinal cord injury | Concussion and oedema of cervical spinal cord | S14.0 | n=N/A*  * This study did not specify the number of patients that fell under each comparator group, but the total sample size was n=260 (19.5% were applicable for this study’s analysis) | 76.2 | - | 14.3 | 98.8 | - | - | - |  |
|  |  | Other unspecified injuries of cervical spinal cord | S14.1 |  | 100.0 | - | 22.9 | 100.0 | - | - | - |  |
|  |  | Concussion and oedema of thoracic spinal cord | S24.0 |  | 91.7 | - | 10.5 | 0.998 | - | - | - |  |
|  |  | Other and unspecified injuries of thoracic spinal cord | S24.1 |  | 94.1 | - | 15.2 | 99.8 | - | - | - |  |
|  |  | Concussion and oedema of lumbar spinal cord | S34.0 |  | 33.3 | - | 0.9 | 99.5 | - | - | - |  |
|  |  | Other injury of lumbar spinal cord | S34.1 |  | 100.0 | - | 6.7 | 100.0 | - | - | - |  |
|  |  | Injury of cauda equina | S34.3 |  | 85.7 | - | 5.7 | 99.8 | - | - | - |  |
|  |  | Sequelae of injury of spinal cord | T91.3 |  | 89.7 | - | 33.3 | 99.1 | - | - | - |  |
| **Abuse** | | | | | | | | | | | | |
| **(Rasooly et al., 2023)** | Child physical abuse, assault, and other maltreatment | Identifying cases where abuse was considered | T74.12; Y09; Y07; T76.12; Y04; Y08.89XA | T74.12 (n=38), Y09 (n=14), Y07 (n=43), T76.12 (n=140), Y04 (n=23), Y08.89XA (n=5) | 97 (86-100); 93 (66-100); 86 (72-95); 94 (88-97); 91 (72-99); 81 (58-95) | - | - | - | - | - | 0.63 |  |
|  |  | Identifying cases where there was definitely or likely abuse |  |  | 89 (75-97); 86 (57-98); 72 (56-85); 59 (50-67); 52 (31-73); 24 (8-47) | - | - | - | - | - |  |  |
| **(Hughes Garza et al., 2021)** | Child maltreatment and physical abuse | Overall | T74.12, T74.4, T74.92, T76.12, T76.92, Y07, Y09 | T74.12 (n=10)  T74.4 (n=1)  T74.92 (n=2)  T76.12 (n=56)  T76.92 (n=7)  Y07 (n=9)  Y09 (n=3) | 76.0 (65.0-84.9) | 44.6 (38.1-51.9) | 31.8 (25.2-38.9) | 84.6 (76.9-90.4) | - | - | - | ^a^ Outpatients included those seen in the outpatient child abuse clinic and those seen in the emergency department and not admitted.  ^b^ Patients classified as inpatients included those admitted to any hospital ward or intensive care unit. |
| **(McKenzie et al., 2011)** | Child maltreatment and assault, sexual abuse, and physical abuse | Physical abuse | T74, Z61.6,  X85-Y09 | T74 (n=467) | - | - | 72.6 | 90.8 | - | 0.818 | - |  |
|  |  | Other or unspecified abuse |  | Z61.6, X85-Y09 (n=467) | - | - | 62.5 | 87.3 | - |  | - |  |
| **Brain Injuries** | | | | | | | | | | | | |
| **(McChesney et al., 2017)** | Brain injury outcomes (skull fractures, concussions, cerebral edema, traumatic brain injuries, hemorrhage, other intracranial injuries) | Codes compared to chart review uniformly | S02.000-S02.001, S02.100-S02.101, S02.300, S02.800, S02.890, S02.900-S02.901, S06.010, S06.020, S06.090, S06.190, S06.200, S06.210, S06.230, S06.290, S06.340, S06.35, S06.390, S06.4, S06.400-S06.401, S06.420, S06.500, S06.510, S06.530, S06.590-S06.591, S06.6, S06.600, S06.610, S06.630, S06.640, S06.690, S06.691, S06.800, S06.810, S06.890, S06.9, S06.900, S06.910, S06.990-S06.991, S09.9 | S02.000 (n=19), S02.001 (n=3), S02.100 (n=14), S02.101 (n=10), S02.300 (n=71), S02.800 (n=1), S02.890 (n=6), S02.900 (n=18), S02.901 (n=1), S06.010 (n=126), S06.020 (n=1), S06.090 (n=77), S06.190 (n=1), S06.200 (n=3), S06.210 (n=2), S06.230 (n=1), S06.290 (n=5), S06.340 (n=1), S06.35 (n=1), S06.390 (n=2), S06.4 (n=2), S06.400 (n=15), S06.401 (n=1), S06.420 (n=1), S06.500 (n=13), S06.510 (n=5), S06.530 (n=1), S06.590 (n=2), S06.591 (n=1), S06.6 (n=2), S06.600 (n=3), S06.610 (n=1), S06.630 (n=1), S06.640 (n=1), S06.690 (n=2), S06.691 (n=1), S06.800 (n=10), S06.810 (n=7), S06.890 (n=3), S06.9 (n=16), S06.900 (n=87), S06.910 (n=19), S06.990 (n=23), S06.991 (n=1), S09.9 (n=1145) | 60.6 (58.2-63.0) | 92.8 (92.0-93.5) | 72.9 (70.5-75.3) | 88.0 (87.1-88.8) | - | - | - |  |
| **(Warwick et al., 2020)** | Outcomes for skull fractures, intracranial injuries, and shaken infant syndrome | All inpatient samples of traumatic brain injury (TBI)-related records | S02.0-S02.1, S02.8, S02.91, S04.02, S04.03, S04.04, S06, S07.1, T74.4 | S02.0-S02.1, S02.8, S02.91, S04.02, S04.03, S04.04, S06, S07.1, T74.4 (n=196) | 74.0 (67.9–80.1) | - | - | - | - | - | - |  |
| **(Peterson et al., 2021)** | Unspecified injuries of head | Level of certainty^o^  Maryland:  High | S09.90 | n=130 | 37.0 | - | - | - | - | - | - | ^l^A level of certainty of the TBI diagnosis was assigned to each medical record (contains ICD-10-CM diagnosis codes), based on the number and type of symptoms and imaging results present in the record. |
|  |  | Maryland:  All levels of certainty (high, medium, low) |  | n=246 | 70.1 | - | - | - | - | - | - |  |
|  |  | Kentucky:  High |  | n=103 | 26.8 | - | - | - | - | - | - |  |
|  |  | Kentucky: All levels of certainty (high, medium, low) |  | n=232 | 60.3 | - | - | - | - | - | - |  |
|  |  | Colorado:  High |  | n=156 | 40.8 | - | - | - | - | - | - |  |
|  |  | Colorado:  All levels of certainty (high, medium, low) |  | n=273 | 71.5 | - | - | - | - | - | - |  |
|  |  | Massachusetts:  High |  | n=87 | 22.7 | - | - | - | - | - | - |  |
|  |  | Massachusetts:  All levels of certainty (high, medium, low) |  | n=282 | 73.7 | - | - | - | - | - | - |  |
| **(Shehab et al., 2019)** | Traumatic brain injuries | Any acute care encounter, code in any position^m^ | S065X0A | n=20 | 90.0 | - | - | - | - | - | - | ^m^Refers to the diagnosis position (in patient charts) of the code |
|  |  |  | S066X0A | n=9 | 100.0 | - | - | - | - | - | - |  |
|  |  |  | S065X9A | n=5 | 100.0 | - | - | - | - | - | - |  |
|  |  |  | S066X9A | n=3 | 33.3 | - | - | - | - | - | - |  |
|  |  | Adverse effect and poisoning codes | T45515A | n=91 | 71.4 (61.0-80.4) | - | 6.8 (6.0-7.8) | - | - | - | - |  |
| **(Peng et al., 2018)** | Open wounds on head, wrists, and hands; fractures, sprains, and strains of joints and ligaments at ankle and foot level | Open wound of head | S01 | n=15 | 93 (68-100) | - | 81 (57-96) | - | - | - | - | Study outcomes split into outcomes for brain injuries, hand and wrist injuries, and lower extremities injuries |
| **Poisoning** | | | | | | | | | | | | |
| **(Chiang et al., 2022)** | Toxic effect of carbon monoxide (i.e., poisoning) | T58 as primary diagnosis | T58 | n=119 | 97.9 (92.8-99.8) | 87.9 (82.3-92.3) | 81.2 (72.9-87.8) | 98.8 (95.6-99.9) | - | - | - |  |
|  |  | T58 as primary or secondary diagnosis |  | n=143 | 80.2 (72.3-86.6) | 91.9 (86.3-95.7) | 89.7 (82.8-94.6) | 84 (77.4-89.2) | - | - | - |  |
|  |  | T58 as primary, secondary, or tertiary diagnosis |  | n=147 | 78.1 (70.2-84.7) | 93 (87.4-96.6) | 91.5 (84.8-95.8) | 81.5 (74.6-87.1) | - | - | - |  |
|  |  | T58 in any field of diagnosis |  | n=151 | 76.6 (66.8-83.2) | 95.5 (90.5-98.3) | 94.9 (89.2-98.1) | 79.0 (71.9-85.0) | - | - | - |  |
| **(Cheng et al., 2021)** | Poisoning from drugs and biological substances | Poisoning by systemic antibiotics | T36 | n=783 | 60.3 | - | - | - | - | - | - |  |
|  |  | Poisoning by other systemic anti-infectives and antiparasites | T37 | n=191 | 60.2 | - | - | - | - | - | - |  |
|  |  | Poisoning by nonopioid analgesics, antipyretics and antirheumatics | T39 | n=64 | 32.8 | - | - | - | - | - | - |  |
|  |  | Poisoning by antiepileptic, sedative-hypnotic and antiparkinsonism drugs | T42 | n=245 | 55.9 | - | - | - | - | - | - |  |
|  |  | Poisoning by diuretics and other and unspecified drugs, medicaments and biological substances | T50 | n=21 | 52.4 | - | - | - | - | - | - |  |
| **(Green et al., 2017)** | Poisoning by opioids, other synthetic narcotics, and psychodysleptics (intentional or unintentional) | Identification of opioid overdoses and poisonings among all events related to opioids (e.g., overdoses and opioid-related adverse effects) ^j^ | T40.1-T40.3, X42, X62, Y12 | T40.1 (n=1)  T40.2 (n=3)  T40.3 (n=1)  X42 (n=35)  X62 (n=5)  Y12 (n=1) | 84 | - | - | - | - | - | - | ^j^About 61% of the single or poly-drug opioid-related events were unintentional overdose events, about 22% were intentional overdoses (e.g., they had some indicator that the person was attempting suicide), and 16% of events identified using poisoning codes were classified as opioid-related adverse effects that were not overdoses or poisonings |
|  |  | Identification of opioid overdoses and poisonings among all non-anesthesia-related events identified (i.e., not just opioid-related events) |  | T40.1 (n=1)  T40.2 (n=3)  T40.3 (n=1)  X42 (n=37)  X62 (n=5) | 81 | - | - | - | - | - | - |  |
| **(Henderson et al., 2006)** | Femur fractures; transport incident injuries; accidental poisoning and exposure to noxious substances | Poisoning and toxic effects: 1998-1999 | X40-X49 | This study did not specify the portion of the sample size that fell under this comparator group, but the total sample size was n<14635 (an unspecified number were applicable for this study’s analysis) | 67 (50-84%) | - | 83 (68-98) | - | - | 0.74 | - | Study outcomes split into outcomes for lower extremities injuries, transport and pedestrian injuries, and poisoning |
|  |  | Poisoning and toxic effects: 2000-2001 |  |  | 71 (55-87) | - | 76 (60-91) | - | - | 0.73 | - |  |
| **Lower Extremities Injuries** | | | | | | | | | | | | |
| **(Seltzer et al., 2022)** | Ankle fracture outcomes | Medial malleolus fracture | S82.5 | n=10 | 50 (19-81) | 98 (92-100) | 71 (29-96) | 94 (88-98) | - | 0.26 | - |  |
|  |  | Lateral malleolus fracture | S82.6 | n=23 | 91 (72-99) | 54 (42-66) | 38 (25-52) | 95 (84-99) | - |  | - |  |
|  |  | Other fracture of the fibula | S82.83 | n=23 | 0 (0-15) | 99 (93-100) | 0 (0-97) | 76 (66-84) | - |  | - |  |
|  |  | Bimalleolar fracture | S82.84 | n=8 | 75 (35-97) | 87 (78-93) | 33 (13-59) | 97 (91-100) | - |  | - |  |
|  |  | Trimalleolar fracture | S82.85 | n=7 | 43 (10-82) | 97 (91-99) | 50 (12-88) | 96 (88-99) | - |  | - |  |
|  |  | Pilon fracture | S82.87 | n=4 | 50 (7-93) | 97 (91-99) | 40 (5-85) | 98 (92-100) | - |  | - |  |
|  |  | Other fracture of lower leg | S82.89 | n=21 | 5 (0-24) | 95 (87-99) | 20 (1-73) | 78 (68-86) | - |  | - |  |
| **(Schneble et al., 2020)** | Proximal femur fractures outcomes | Inter-rater reliability:  general code that identified the fractured region | Proximal femur fractures ICD10 codes (unspecified) | This study did not specify the codes, or portion of the sample size, that fell under each comparator group, but the total sample size was n=196 (105 were applicable for this study’s analysis) | - | - | - | - | 0.593 (0.512-0.683) | - | - |  |
|  |  | Inter-rater reliability:  exact code that identified the fractured region |  |  | - | - | - | - | 0.313 (0.243-0.383) | - | - |  |
|  |  | General code that identified the fractured region: correct code vs. EMR |  |  | - | - | - | - | - | 0.485 (0.373-0.597) | - |  |
|  |  | General code that identified the fractured region: correct code vs. billing code |  |  | - | - | - | - | - | 0.604 (0.493-0.716) | - |  |
|  |  | Exact code that identified the fractured region: at least correct EMR code present |  |  | - | - | - | - | - | 0.294 (0.208-0.380) | - |  |
|  |  | Exact code that identified the fractured region: only correct EMR code(s) present |  |  | - | - | - | - | - | 0.255 (0.173-0.337) | - |  |
| **(Thuy Trinh et al., 2018)** | Hip fractures outcomes | By episode ^h^:  Principle diagnosis | S72.00-S72.05, S72.08, S72.10-S72.11, S72.2 | S72.00-S72.05  S72.08  S72.10-S72.11  S72.2 (n=864) | 68 (66–70) | - | 93 (92–94) | - | - | - | - | ^h^all episodes with a hip fracture (HF) code in either primary diagnosis (PD) or additional diagnoses (ADs) were counted.  ^i^each admission with a HF coded as the PD or an AD was counted as one HF, irrespective of the number of HF episodes coded during that admission since the subsequent codes were likely to be the coding of multiple treatments for the same HF |
|  |  | By episode ^h^:  Additional diagnosis |  | S72.00-S72.05  S72.08  S72.10-S72.11  S72.2 (n=276) | 6.2 (4.7–7.7) | - | 2.7 (2.1–3.3) | - | - | - | - |  |
|  |  | By episode ^h^:  All diagnoses |  | S72.00-S72.05  S72.08  S72.10-S72.11  S72.2 (n=1140) | 53 (52–54) | - | 95 (94–96) | - | - | - | - |  |
|  |  | By admission^i^:  Principle diagnosis |  | S72.00-S72.05  S72.08  S72.10-S72.11  S72.2 (n=743) | 80 (79–81) | - | 93 (92–94) | - | - | - | - |  |
|  |  | By admission^i^:  Additional diagnosis |  | S72.00-S72.05  S72.08  S72.10-S72.11  S72.2 (n=84) | 10 (6.7–13) | - | 1.3 (0.9–1.7) | - | - | - | - |  |
|  |  | By admission^i^:  All diagnoses |  | S72.00-S72.05  S72.08  S72.10-S72.11  S72.2 (n=827) | 72 (70–74) | - | 94 (93–95) | - | - | - | - |  |
| **(Peng et al., 2018)** | Open wounds on head, wrists, and hands; fractures, sprains, and strains of joints and ligaments at ankle and foot level | Fracture of lower leg, including ankle | S82 | n=15 | 87 (60-98) | - | 87 (60-98) | - | - | - | - | Study outcomes split into outcomes for brain injuries, hand and wrist injuries, and lower extremities injuries |
|  |  | Dislocation, sprain and strain of joints and ligaments at ankle and foot level | S93 | n=15 | 100 (78-100) | - | 94 (70-100) | - | - | - | - |  |
| **(Henderson et al., 2006)** | Femur fractures; transport incident injuries; accidental poisoning and exposure to noxious substances | Hip fractures:  1998-1999 | S720-S721 | This study did not specify the portion of the sample size that fell under this comparator group, but the total sample size was n<14635 (an unspecified number were applicable for this study’s analysis) | 100 | - | 94 (85-100) | - | - | 0.97 | - | Study outcomes split into outcomes for lower extremities injuries, transport and pedestrian injuries, and poisoning |
|  |  | Hip fractures: 2000-2001 |  |  | 91 (83-99) | - | 95 (89-100) | - | - | 0.93 | - |  |
| **Multiple (Total Body) Injury Types Reported** | | | | | | | | | | | | |
| **(Watzlaf et al., 2007)** | External-cause injuries to any body part outcomes; burns and corrosions; frostbite; maltreatment and abuse |  | S00-T34, T51-T65, T74, T76 | S00-T34, T51-T65, T74, T76 (n=23) | - | - | - | - | - | 0.15 | - |  |
| **(Miller et al., 2022)** | Firearm injuries (accidental and intentional) as assault to others and self-harm | Unintentional | W320XX-W321XX, W330XX-W3309X, W3309XW3313X, W3319X, W3400X, W3409X-W3410X, W3419X, X72XXX, X730XX-X732XX, X738XXX739XX, X748XX-X749XX, Y384X1-Y384X3, X93XXX, X940XX-X942XX, X948XX-X949XX, X958XX-X959XX, Y22XXX, Y230XX-Y233XX, Y238XX-Y239XX, Y248XX-Y249XX, Y35001-Y35003, Y35009, Y35011-Y35013, Y35019, Y35021-Y35023, Y35029, Y35031-Y35033, Y35039, Y35091- Y35093, Y35099 | W320XX-W321XX, W330XX-W3309X, W3309XW3313X, W3319X, W3400X, W3409X-W3410X, W3419X (n=432) | 34.3 | - | 88.1 | - | - | - | - |  |
|  |  | Assault |  | X93XXX, X940XX-X942XX, X948XX-X949XX, X958XX-X959XX, Y384X1-Y384X3 (n=581) | 95.5 | - | 66.3 | - | - | - | - |  |
|  |  | Legal intervention codes |  | Y35001-Y35003, Y35009, Y35011-Y35013, Y35019, Y35021-Y35023, Y35029, Y35031-Y35033, Y35039, Y35091- Y35093, Y35099 (n=32) | 93.8 | - | 69.8 | - | - | - | - |  |
|  |  | Suicide/self-harm (n=119) |  | X72XXX, X730XX-X732XX, X738XXX739XX, X748XX-X749XX (n=119) | 93.3 | - | 89.5% | - | - | - | - |  |
|  |  | Undetermined (n=55) |  | Y22XXX, Y230XX-Y233XX, Y238XX-Y239XX, Y248XX-Y249XX (n=55) | 5.5 | - | 5.0 | - | - | - | - |  |
| **(Brown et al., 2023)** | Injuries of the head and neck outcomes and mechanisms of physical abuse, assault, and other related injury mechanisms | Codes compared to chart review uniformly | S00-S09, T04.0, T06.0, T74.4, T74.1, T74.9, T76.1, T76.9, Y00, Y01, Y04, Y07-Y09, Y29, Y30, Y33 | S00 (n=216), S01(n=43), S02 (n=128), S03 (n=0), S04 (n=1), S05 (n=15), S06 (n=327), S07 (n=0), S08 (n=0)  S09 (n=100), T04.0 (n=0), T06.0 (n=0), T74.4 (n=14), T74.1(n=150), T74.9 (n=54), T76.1 (n=195), T76.9 (n=57), Y00 (n=2), Y01 (n=0), Y04 (n=15), Y07 (n=69), Y08 (n=15), Y09 (n=52), Y29 (n=1), Y30 (n=1), Y33 (n=10) | 92.6 (90.1−95.1) | 94.4 (93.4−95.3) | 76.1 (72.5−79.8) | 98.5 (98.0−99.0) | - | - | - |  |
| **(Asadi et al., 2022)** | Trauma code outcomes (codes not specified further) | Agreement between Coder 1 and the primary coder on the coding of the nature of trauma in the research units | Trauma (mechanism) coding injury ICD10 codes (unspecified) | This study did not specify the codes, or portion of the sample size, that fell under each comparator group, but the total sample size was n=591 | - | - | - | - | - | 0.75 | - |  |
|  |  | Agreement between Coder 2 and the primary coder on the coding of the nature of trauma in the research units |  |  | - | - | - | - | - | 0.77 | - |  |
| **Hand and Wrist Injuries** | | | | | | | | | | | | |
| **(Peng et al., 2018)** | Open wounds on head, wrists, and hands; fractures, sprains, and strains of joints and ligaments at ankle and foot level | Open wound of wrist and hand | S61 | n=24 | 92 (73-99) | - | 96 (78-100) | - | - | - | - | Study outcomes split into outcomes for brain injuries, hand and wrist injuries, and lower extremities injuries |

Abbreviations: electronic medical record (EMR), spinal cord injury (SCI), traumatic spinal cord injury (TSCI)

*Codes included are reported as grouped together in the papers’ results/outcomes
