# Supplementary material for: Validity and reliability International Classification of Diseases-10 codes for all forms of injury: A systematic review
Source: PLoS One. 2024 Feb 29;19(2):e0298411. doi: 10.1371/journal.pone.0298411 (PMC10903801; doi:10.1371/journal.pone.0298411)
Supplement: S2 Table — (DOCX) [file pone.0298411.s008.docx]

**Table S2. Risk of bias assessment summary**

|  | Question 1 | Question 2 | Question 3 | Question 4 | Question 5 | Question 6 | Question 7 | Question 8 | Question 9 | Question 10 | Question 11 | Question 12 | Question 13 | Question 14 | Total Score | Number of N/A Items | Overall study quality assessment |
| --- | --- | --- | --- | --- | --- | --- | --- | --- | --- | --- | --- | --- | --- | --- | --- | --- | --- |
| Furlan & Fehlings (2011) | 1 | 1 | 1 | NA | 1 | 1 | 1 | 1 | 1 | U | U | 1 | NA | NA | 11.5 | 3 | High |
| Randall et al., (2017) | 1 | 1 | 1 | NA | 1 | 1 | 1 | 1 | 1 | 1 | 1 | 1 | NA | NA | 13 | 3 | High |
| Rasooly et al., (2023) | 1 | 1 | 1 | NA | 1 | 1 | 1 | 1 | 1 | U | U | 1 | 1 | 1 | 11.5 | 1 | High |
| McChesney-Corbeil et al., (2017) | 1 | 1 | 1 | NA | 1 | 1 | 1 | 1 | 1 | U | U | 1 | NA | 1 | 11.5 | 2 | High |
| Sveticic et al., (2020) | 1 | 1 | 1 | NA | 1 | 1 | 1 | 1 | 1 | U | U | 1 | 1 | 1 | 11.5 | 1 | High |
| Hughes Garza et al., (2021) | 1 | 1 | 1 | NA | 1 | 1 | 1 | 1 | 1 | U | 1 | 1 | NA | NA | 12.5 | 3 | High |
| Gabella et al., (2022) | 1 | 1 | 1 | NA | 1 | 1 | 1 | 1 | 1 | 1 | 1 | 1 | 1 | 1 | 13 | 1 | High |
| Chiang et al., (2022) | 1 | 1 | 1 | NA | 1 | 1 | 1 | 1 | 1 | U | U | 1 | NA | NA | 11.5 | 3 | High |
| Miller et al., (2022) | 1 | 1 | 1 | NA | 1 | 1 | 1 | 1 | 1 | U | U | 1 | NA | NA | 11.5 | 3 | High |
| Seltzer et al., (2022) | 1 | 1 | 1 | NA | 1 | 1 | 1 | 1 | 1 | U | U | 1 | 1 | 1 | 11.5 | 1 | High |
| Karkhaneh et al., (2012) | 1 | 1 | 1 | NA | 1 | 1 | 1 | 1 | 0 | 1 | 1 | 1 | 1 | NA | 12 | 2 | High |
| Brown et al., (2023) | 1 | 1 | 1 | NA | 1 | 0 | 1 | 1 | 1 | 0 | U | 1 | 1 | 1 | 10 | 1 | Medium |
| Thuy Trinh et al., (2018) | 1 | 1 | 1 | NA | 1 | 1 | 1 | 1 | 1 | U | U | 1 | 1 | 1 | 11.5 | 1 | High |
| Warwick et al., (2020) | 1 | 1 | 1 | NA | 1 | 1 | 1 | 1 | 1 | 1 | U | 1 | NA | NA | 12 | 3 | High |
| Hagen et al., (2009) | 1 | 1 | 1 | NA | 1 | 1 | 1 | 1 | 0 | U | U | 1 | NA | 1 | 10.5 | 2 | Medium |
| McKenzie et al., (2011) | 1 | 1 | 1 | NA | 1 | 1 | 1 | 1 | 1 | 1 | 1 | 1 | NA | 0 | 12 | 2 | High |
| Green et al., (2017) | 1 | 1 | 1 | NA | 1 | 1 | 1 | 1 | 1 | 1 | U | 1 | 1 | 1 | 12 | 1 | High |
| Schneble et al., (2020) | 1 | 1 | 1 | NA | 1 | 1 | 1 | 1 | 1 | U | 1 | 1 | NA | NA | 12.5 | 3 | High |
| Asadi et al., (2022) | 1 | 1 | 1 | NA | 1 | 1 | 1 | 1 | 1 | 1 | 1 | 1 | NA | NA | 13 | 3 | High |
| Peng et al., (2018) | 1 | 1 | 1 | NA | 1 | 1 | 1 | 1 | 1 | 1 | 1 | 1 | NA | NA | 13 | 3 | High |
| Welk et al., (2014) | 1 | 1 | 1 | NA | 1 | 1 | 1 | 1 | 1 | 1 | 1 | 1 | NA | NA | 13 | 3 | High |
| Hansen et al., (2021) | 1 | 1 | 1 | NA | 1 | 1 | 1 | 1 | 1 | 1 | 1 | 1 | NA | NA | 13 | 3 | High |
| Peterson et al., (2021) | 1 | 1 | 1 | NA | 1 | 1 | 1 | 1 | 1 | U | U | 1 | 1 | 0 | 10.5 | 1 | Medium |
| Henderson et al., (2006) | 1 | 1 | 1 | NA | 1 | 1 | 1 | 1 | 1 | U | 0 | 1 | NA | 1 | 10.5 | 2 | Medium |
| Watzlaf et al., (2007) | 1 | 1 | 1 | NA | 1 | 1 | 1 | 1 | 0 | U | U | 1 | NA | NA | 10.5 | 3 | Medium |
| Cheng et al., (2021) | 1 | 1 | 1 | NA | 1 | 1 | 1 | 1 | 1 | 1 | 1 | 1 | NA | NA | 13 | 3 | High |
| Shehab et al., (2019) | 1 | 1 | 1 | NA | 1 | 1 | 1 | 1 | 1 | 1 | 1 | 1 | 1 | 1 | 13 | 1 | High |

1=Yes; 0=No; 0=Unclear; N/A=Not Applicable (N/A)
