# Supplementary material for: Validity and reliability International Classification of Diseases-10 codes for all forms of injury: A systematic review
Source: PLoS One. 2024 Feb 29;19(2):e0298411. doi: 10.1371/journal.pone.0298411 (PMC10903801; doi:10.1371/journal.pone.0298411)
Supplement: S3 Table — (DOCX) [file pone.0298411.s009.docx]

**Table S3.** **Detailed breakdown of all codes examined for each study.** Code descriptions were named from the official International Statistical Classification of Diseases and Related Health Problems, Tenth Revision, from the Canadian Institute for Health Information, 2018. Other sources were used for codes not listed in this source.

| Study | Evaluated Code(s) | Code Definition |
| --- | --- | --- |
| **(Furlan & Fehlings, 2011)** | S06.400 | Epidural haemorrhage |
|  | S12.1 | Fracture of second cervical vertebra |
|  | S12.900 | Fracture of neck, part unspecified, closed |
|  | S14.10 | Complete lesion of cervical spinal cord |
|  | S22.000 | Fracture of thoracic vertebra |
|  | S23.1 | Dislocation of thoracic vertebra |
|  | S24.10 | Complete lesion of thoracic spinal cord |
|  | S32.000 | Fracture of lumbar vertebra |
|  | S32.100 | Fracture of sacrum, closed |
|  | T06.1 | Injuries of nerves and spinal cord involving other multiple body regions |
| **(Randall et al., 2017)** | X40-X49 | Accidental poisoning by and exposure to noxious substances |
|  | X60-X84 | Intentional self-harm |
|  | Y10-Y34 | Event of undetermined intent |
| **(Rasooly et al., 2023)** | T74.12 | Child physical abuse, confirmed (2023 ICD-10-CM codes, 2023) |
|  | T76.12 | Child physical abuse, suspected (2023 ICD-10-CM codes, 2023) |
|  | Y04 | Assault by bodily force |
|  | Y07 | Other maltreatment |
|  | Y08.89XA | Assault by other specified means, initial encounter (2023 ICD-10-CM codes, 2023) |
|  | Y09 | Assault by unspecified means |
| **(McChesney-Corbeil et al., 2017)** | S02.000 | Fracture of vault of skull, closed |
|  | S02.001 | Fracture of vault of skull, open |
|  | S02.100 | Fracture of base of skull, closed |
|  | S02.101 | Fracture of base of skull, open |
|  | S02.300 | Fracture of orbital floor, closed |
|  | S02.800 | Fracture of alveolus, closed |
|  | S02.890 | Fractures of other unspecified skull and facial bones, closed |
|  | S02.900 | Fracture of skull and facial bones, part unspecified, closed |
|  | S02.901 | Fracture of skull and facial bones, part unspecified, open |
|  | S06.010, S06.020, S06.090 | Concussion without loss of consciousness (2023 ICD-10-CM codes, 2023) |
|  | S06.190 | Traumatic cerebral edema without loss of consciousness (2023 ICD-10-CM codes, 2023) |
|  | S06.200, S06.210, S06.230, S06.290 | Diffuse traumatic brain injury without loss of consciousness (2023 ICD-10-CM codes, 2023) |
|  | S06.340 | Traumatic hemorrhage of right cerebrum without loss of consciousness (2023 ICD-10-CM codes, 2023) |
|  | S06.35 | Focal brain injury without open intracranial wound |
|  | S06.390 | Focal brain injury with loss of consciousness of unspecified duration, without open intracranial wound (McChesney et al., 2017) |
|  | S06.4 | Epidural haemorrhage |
|  | S06.400, S06.420 | Epidural hemorrhage without loss of consciousness (2023 ICD-10-CM codes, 2023) |
|  | S06.401 | Epidural hemorrhage with loss of consciousness of 30 minutes or less (2023 ICD-10-CM codes, 2023) |
|  | S06.500, S06.510, S06.530, S06.590 | Traumatic subdural hemorrhage without loss of consciousness (2023 ICD-10-CM codes, 2023) |
|  | S06.591 | Traumatic subdural hemorrhage with loss of consciousness of 30 minutes or less (2023 ICD-10-CM codes, 2023) |
|  | S06.6 | Traumatic subarachnoid haemorrhage |
|  | S06.800 | Other specified intracranial injuries (2023 ICD-10-CM codes, 2023) |
|  | S06.810 | Injury of right internal carotid artery, intracranial portion, not elsewhere classified without loss of consciousness (2023 ICD-10-CM codes, 2023) |
|  | S06.890 | Other specified intracranial injury without loss of consciousness (2023 ICD-10-CM codes, 2023) |
|  | S06.9 | Intracranial injury, unspecified |
|  | S09.9 | Unspecified injury of head |
| **(Sveticic et al., 2020)** | X84 | Intentional self-harm by unspecified means |
| **(Hughes Garza et al., 2021)** | T74.12 | Child physical abuse, confirmed (2023 ICD-10-CM codes, 2023) |
|  | T74.4 | Shaken infant syndrome (2023 ICD-10-CM codes, 2023) |
|  | T74.92 | Unspecified child maltreatment, confirmed (2023 ICD-10-CM codes, 2023) |
|  | T76.12 | Child physical abuse, suspected (2023 ICD-10-CM codes, 2023) |
|  | T76.92 | Unspecified child maltreatment, suspected (2023 ICD-10-CM codes, 2023) |
|  | Y07 | Other maltreatment |
|  | Y09 | Assault by unspecified means |
| **(Gabella et al., 2022)** | T14.91 | Suicide attempt (2023 ICD-10-CM codes, 2023) |
|  | T36–T50 | Poisoning by drugs, medicaments and biological substances |
|  | T51–T65 | Toxic effects of substances chiefly nonmedical as to source |
|  | T71 | Asphyxiation |
|  | X71 | Intentional self-harm by drowning and submersion |
|  | X72 | Intentional self-harm by handgun discharge |
|  | X73 | Intentional self-harm by rifle, shotgun and larger firearm discharge |
|  | X74 | Intentional self-harm by other and unspecified firearm discharge |
|  | X75 | Intentional self-harm by explosive material |
|  | X76 | Intentional self-harm by smoke, fire, and flames |
|  | X77 | Intentional self-harm by steam, hot vapours and hot objects |
|  | X78 | Intentional self-harm by sharp object |
|  | X79 | Intentional self-harm by blunt object |
|  | X80 | Intentional self-harm by jumping from a high place |
|  | X81 | Intentional self-harm by jumping or lying before moving object |
|  | X82 | Intentional self-harm by crashing of motor vehicle |
|  | X83 | Intentional self-harm by other specified means |
| **(Chiang et al., 2022)** | T58 | Toxic effect of carbon monoxide |
| **(Miller et al., 2022)** | W320XX | Accidental handgun discharge (2023 ICD-10-CM codes, 2023) |
|  | W321XX | Accidental handgun malfunction (2023 ICD-10-CM codes, 2023) |
|  | W330XX-W3309X, W3309X | Accidental rifle, shotgun and larger firearm discharge (2023 ICD-10-CM codes, 2023) |
|  | W3313X, W3319X | Accidental rifle, shotgun and larger firearm malfunction (2023 ICD-10-CM codes, 2023) |
|  | W3400X | Accidental discharge from unspecified firearms or gun (2023 ICD-10-CM codes, 2023) |
|  | W3409X | Accidental discharge from other specified firearms (2023 ICD-10-CM codes, 2023) |
|  | W3410X | Accidental malfunction from unspecified firearms or gun (2023 ICD-10-CM codes, 2023) |
|  | W3419X | Accidental malfunction from other specified firearms (2023 ICD-10-CM codes, 2023) |
|  | X72XXX | Intentional self-harm by handgun discharge |
|  | X730XX | Intentional self-harm by shotgun discharge (2023 ICD-10-CM codes, 2023) |
|  | X731XX | Intentional self-harm by hunting rifle discharge (2023 ICD-10-CM codes, 2023) |
|  | X732XX | Intentional self-harm by machine gun discharge (2023 ICD-10-CM codes, 2023) |
|  | X738XX | Intentional self-harm by other larger firearm discharge (2023 ICD-10-CM codes, 2023) |
|  | X739XX | Intentional self-harm by unspecified larger firearm discharge (2023 ICD-10-CM codes, 2023) |
|  | X748XX | Intentional self-harm by other firearm discharge (2023 ICD-10-CM codes, 2023) |
|  | X749XX | Intentional self-harm by unspecified firearm discharge (2023 ICD-10-CM codes, 2023) |
|  | X93XXX | Assault by handgun discharge |
|  | X940XX | Assault by shotgun (2023 ICD-10-CM codes, 2023) |
|  | X941XX | Assault by hunting rifle (2023 ICD-10-CM codes, 2023) |
|  | X942XX | Assault by machine gun (2023 ICD-10-CM codes, 2023) |
|  | X948XX | Assault by other larger firearm discharge (2023 ICD-10-CM codes, 2023) |
|  | X949XX | Assault by unspecified larger firearm discharge (2023 ICD-10-CM codes, 2023) |
|  | X958XX | Assault by other firearm discharge (2023 ICD-10-CM codes, 2023) |
|  | X959XX | Assault by unspecified firearm discharge (2023 ICD-10-CM codes, 2023) |
|  | Y22XX | Handgun discharge, undetermined intent |
|  | Y230XX- | Shotgun discharge, undetermined intent (2023 ICD-10-CM codes, 2023) |
|  | Y231XX | Hunting rifle discharge, undetermined intent (2023 ICD-10-CM codes, 2023) |
|  | Y232XX | Military firearm discharge, undetermined intent (2023 ICD-10-CM codes, 2023) |
|  | Y233XX | Machine gun discharge, undetermined intent (2023 ICD-10-CM codes, 2023) |
|  | Y238XX | Other larger firearm discharge, undetermined intent (2023 ICD-10-CM codes, 2023) |
|  | Y239XX | Unspecified larger firearm discharge, undetermined intent (2023 ICD-10-CM codes, 2023) |
|  | Y248XX | Other firearm discharge, undetermined intent (2023 ICD-10-CM codes, 2023) |
|  | Y249XX | Unspecified firearm discharge, undetermined intent (2023 ICD-10-CM codes, 2023) |
|  | Y35001 | Legal intervention involving unspecified firearm discharge, law enforcement official injured (2023 ICD-10-CM codes, 2023) |
|  | Y35002 | Legal intervention involving unspecified firearm discharge, bystander injured (2023 ICD-10-CM codes, 2023) |
|  | Y35003 | Legal intervention involving unspecified firearm discharge, suspect injured (2023 ICD-10-CM codes, 2023) |
|  | Y35009 | Legal intervention involving unspecified firearm discharge, unspecified person injured (2023 ICD-10-CM codes, 2023) |
|  | Y35011 | Legal intervention involving injury by machine gun, law enforcement official injured (2023 ICD-10-CM codes, 2023) |
|  | Y35012 | Legal intervention involving injury by machine gun, bystander injured (2023 ICD-10-CM codes, 2023) |
|  | Y35013 | Legal intervention involving injury by machine gun, suspect injured (2023 ICD-10-CM codes, 2023) |
|  | Y35019 | Legal intervention involving injury by machine gun, unspecified person injured (2023 ICD-10-CM codes, 2023) |
|  | Y35021 | Legal intervention involving injury by handgun, law enforcement official injured (2023 ICD-10-CM codes, 2023) |
|  | Y35022 | Legal intervention involving injury by handgun, bystander injured (2023 ICD-10-CM codes, 2023) |
|  | Y35023 | Legal intervention involving injury by handgun, suspect injured (2023 ICD-10-CM codes, 2023) |
|  | Y35029 | Legal intervention involving injury by handgun, unspecified person injured (2023 ICD-10-CM codes, 2023) |
|  | Y35031 | Legal intervention involving injury by rifle pellet, law enforcement official injured (2023 ICD-10-CM codes, 2023) |
|  | Y35032 | Legal intervention involving injury by rifle pellet, bystander injured (2023 ICD-10-CM codes, 2023) |
|  | Y35033 | Legal intervention involving injury by rifle pellet, suspect injured (2023 ICD-10-CM codes, 2023) |
|  | Y35039 | Legal intervention involving injury by rifle pellet, unspecified person injured (2023 ICD-10-CM codes, 2023) |
|  | Y35091 | Legal intervention involving other firearm discharge, law enforcement official injured (2023 ICD-10-CM codes, 2023) |
|  | Y35092 | Legal intervention involving other firearm discharge, bystander injured (2023 ICD-10-CM codes, 2023) |
|  | Y35093 | Legal intervention involving other firearm discharge, suspect injured (2023 ICD-10-CM codes, 2023) |
|  | Y35099 | Legal intervention involving other firearm discharge, unspecified person injured (2023 ICD-10-CM codes, 2023) |
|  | Y384X1 | Terrorism involving firearms, public safety official injured (2023 ICD-10-CM codes, 2023) |
|  | Y384X2 | Terrorism involving firearms, civilian injured (2023 ICD-10-CM codes, 2023) |
|  | Y384X3 | Terrorism involving firearms, terrorist injured (2023 ICD-10-CM codes, 2023) |
| **(Seltzer et al., 2022)** | S82.5 | Fracture of medial malleolus |
|  | S82.6 | Fracture of lateral malleolus |
|  | S82.83 | Fractures of other parts of lower leg |
|  | S82.84 | Bimalleolar fracture of lower leg (2023 ICD-10-CM codes, 2023) |
|  | S82.85 | Trimalleolar fracture of lower leg (2023 ICD-10-CM codes, 2023) |
|  | S82.87 | Pilon fracture of tibia (2023 ICD-10-CM codes, 2023) |
|  | S82.89 | Fracture of ankle NOS |
| **(Karkhaneh et al., 2012)** | Unspecified bicycle and pedestrian physical injuries ICD10 codes | - |
| **(Brown et al., 2023)** | S00-S09 | Injuries to the head |
|  | T04.0 | Crushing injuries involving head with neck |
|  | T06.0 | Injuries of brain and cranial nerves with injuries of nerves and spinal cord at neck level |
|  | T74.1 | Physical abuse |
|  | T74.4 | Shaken infant syndrome (2023 ICD-10-CM codes, 2023) |
|  | T74.9 | Maltreatment syndrome, unspecified |
|  | T76.1 | Physical abuse, suspected (2023 ICD-10-CM codes, 2023) |
|  | T76.9 | Unspecified maltreatment, suspected (2023 ICD-10-CM codes, 2023) |
|  | Y00 | Assault by blunt object |
|  | Y01 | Assault by pushing from high place |
|  | Y04 | Assault by bodily force |
|  | Y07 | Other maltreatment |
|  | Y08 | Assault by other specified means |
|  | Y09 | Assault by unspecified means |
|  | Y29 | Contact with blunt object, undetermined intent |
|  | Y30 | Falling, jumping or pushed from a high place, undetermined intent |
|  | Y33 | Other specified events, undetermined intent |
| **(Thuy Trinh et al., 2018)** | S72.00 | Fracture of upper femoral epiphysis (separation) |
|  | S72.01 | Fracture of base of femoral neck (cervicotrochanteric) |
|  | S72.02 | Fracture of epiphysis (separation) (upper) of femur (2023 ICD-10-CM codes, 2023) |
|  | S72.03 | Midcervical fracture of femur (2023 ICD-10-CM codes, 2023) |
|  | S72.04 | Fracture of base of neck of femur (2023 ICD-10-CM codes, 2023) |
|  | S72.05 | Unspecified fracture of head of femur (2023 ICD-10-CM codes, 2023) |
|  | S72.08 | Other fracture of femoral neck |
|  | S72.10 | Intertrochanteric fracture |
|  | S72.11 | Fracture of greater trochanter of femur (2023 ICD-10-CM codes, 2023) |
|  | S72.2 | Subtrochanteric fracture |
| **(Warwick et al., 2020)** | S02.0 | Fracture of vault of skull |
|  | S02.1 | Fracture of base of skull |
|  | S02.8 | Fracture of other skull and facial bones |
|  | S02.91 | Unspecified fracture of skull (2023 ICD-10-CM codes, 2023) |
|  | S04.02 | Injury of optic chiasm (2023 ICD-10-CM codes, 2023) |
|  | S04.03 | Injury of optic tract and pathways (2023 ICD-10-CM codes, 2023) |
|  | S04.04 | Injury of visual cortex (2023 ICD-10-CM codes, 2023) |
|  | S06 | Intracranial injury |
|  | S07.1 | Crushing injury of skull |
|  | T74.4 | Shaken infant syndrome (2023 ICD-10-CM codes, 2023) |
|  | S14.0 | Concussion and oedema of cervical spinal cord |
|  | S14.1 | Other unspecified injuries of cervical spinal cord |
|  | S24.0 | Concussion and oedema of thoracic spinal cord |
|  | S24.1 | Other and unspecified injuries of thoracic spinal cord |
|  | S34.0 | Concussion and oedema of lumbar spinal cord |
|  | S34.1 | Other injury of lumbar spinal cord |
|  | S34.3 | Injury of cauda equina |
|  | T91.3 | Sequelae of injury of spinal cord |
| **(McKenzie et al., 2011)** | T74 | Maltreatment syndromes |
|  | X85-Y09 | Assault |
|  | Z61.6 | Problems related to alleged physical abuse of child |
| **(Green et al., 2017)** | T40.0 | Poisoning by opium |
|  | T40.1 | Poisoning by heroin |
|  | T40.2 | Poisoning by other opioids |
|  | T40.3 | Poisoning by methadone |
|  | T40.4 | Poisoning by other synthetic narcotics |
|  | X42 | Accidental poisoning by and exposure to narcotics and psychodysleptics [hallucinogens], not elsewhere classified |
|  | X62 | Intentional self-poisoning by and exposure to narcotics and psychodysleptics [hallucinogens], not elsewhere classified |
|  | Y12 | Poisoning by and exposure to narcotics and psychodysleptics [hallucinogens], not elsewhere classified, undetermined intent |
|  | Y45.0 | Opioids and related analgesics |
| **(Schneble et al., 2020)** | Unspecified proximal femur fractures ICD10 codes | - |
| **(Asadi et al., 2022)** | Unspecified trauma coding injury ICD10 codes | - |
| **(Welk et al., 2013)** | S01 | Open wound of head |
|  | S61 | Open wound of wrist and hand |
|  | S82 | Fracture of lower leg, including ankle |
|  | S93 | Dislocation, sprain and strain of joints and ligaments at ankle and foot level |
|  | S14.0 | Concussion and oedema of cervical spinal cord |
|  | S14.1 | Other unspecified injuries of cervical spinal cord |
|  | S24.0 | Concussion and oedema of thoracic spinal cord |
|  | S24.1 | Other and unspecified injuries of thoracic spinal cord |
|  | S34.0 | Concussion and oedema of lumbar spinal cord |
|  | S34.1 | Other injury of lumbar spinal cord |
|  | S34.3 | Injury of cauda equina |
|  | T06.0 | Injuries of brain and cranial nerves with injuries of nerves and spinal cord at neck level |
|  | T06.1 | Injuries of nerves and spinal cord involving other multiple body regions |
| **(Hansen et al., 2021)** | T14.91 | Suicide attempt (2023 ICD-10-CM codes, 2023) |
|  | T36-T50 | Poisoning by drugs, medicaments and biological substances |
|  | T54 | Toxic effect of corrosive substances |
|  | T71 | Asphyxiation |
|  | X78 | Intentional self-harm by sharp object |
|  | X79 | Intentional self-harm by blunt object |
|  | X80 | Intentional self-harm by jumping from a high place |
|  | X83 | Intentional self-harm by other specified means |
| **(Peterson et al., 2021)** | S09.90 | Unspecified injury of head (2023 ICD-10-CM codes, 2023) |
| **(Henderson et al., 2006)** | S720 | Fracture of neck of femur |
|  | S721 | Pertrochanteric fracture |
|  | V40-V49 | Car occupant injured in transport accident |
|  | V50-V59 | Occupant of pick-up truck or van injured in transport accident |
|  | X40-X49 | Accidental poisoning by and exposure to noxious substances |
| **(Watzlaf et al., 2007)** | S00-S09 | Injuries to the head |
|  | S10-S19 | Injuries to the neck |
|  | S20-S29 | Injuries to the thorax |
|  | S30-S39 | Injuries to the abdomen, lower back, lumbar spine and pelvis |
|  | S40-S49 | Injuries to the shoulder and upper arm |
|  | S50-S59 | Injuries to the elbow and forearm |
|  | S60-S69 | Injuries to the wrist and hand |
|  | S70-S79 | Injuries to the hip and thigh |
|  | S80-S89 | Injuries to the knee and lower leg |
|  | S90-S99 | Injuries to the ankle and foot |
|  | T00-T07 | Injuries involving multiple body regions |
|  | T08-T14 | Injuries to unspecified parts of trunk, limb or body region |
|  | T15-T19 | Effects of foreign body entering through natural orifice |
|  | T20-T32 | Burns and corrosions |
|  | T33 | Superficial frostbite |
|  | T34 | Frostbite with tissue necrosis |
|  | T51-T65 | Toxic effects of substances chiefly nonmedical as to source |
|  | T74 | Maltreatment syndromes |
|  | T76 | Adult and child abuse, neglect and other maltreatment, suspected (2023 ICD-10-CM codes, 2023) |
| **(Cheng et al., 2020)** | T36 | Poisoning by systemic antibiotics |
|  | T37 | Poisoning by other systemic anti-infectives and antiparasites |
|  | T39 | Poisoning by nonopioid analgesics, antipyretics and antirheumatics |
|  | T42 | Poisoning by antiepileptic, sedative-hypnotic and antiparkinsonism drugs |
|  | T50 | Poisoning by diuretics and other and unspecified drugs, medicaments and biological substances |
| **(Shehab et al., 2019)** | S065X0A | Traumatic subdural hemorrhage without loss of consciousness, initial encounter (2023 ICD-10-CM codes, 2023) |
|  | S065X9A | Traumatic subdural hemorrhage with loss of consciousness of unspecified duration, initial encounter (2023 ICD-10-CM codes, 2023) |
|  | S066X0A | Traumatic subarachnoid hemorrhage without loss of consciousness, initial encounter (2023 ICD-10-CM codes, 2023) |
|  | S066X9A | Traumatic subarachnoid hemorrhage with loss of consciousness of unspecified duration, initial encounter (2023 ICD-10-CM codes, 2023) |
|  | T45515A | Adverse effect of anticoagulants, initial encounter (2023 ICD-10-CM codes, 2023) |

References:

1. 2023 ICD-10-CM codes [Internet]. ICD10 Data; 2023 [cited 2023 Jul 4]. Available from: <https://www.icd10data.com/ICD10CM/Codes>
2. McChesney CJ, Barlow K, Quan H, Chen G, Wiebe S, Jette N. Validation of a case definition for pediatric brain Injury using administrative data. Can J Neurol Sci. 2017;44(2): 161-169.
