# Supplementary material for: Validity and reliability International Classification of Diseases-10 codes for all forms of injury: A systematic review
Source: PLoS One. 2024 Feb 29;19(2):e0298411. doi: 10.1371/journal.pone.0298411 (PMC10903801; doi:10.1371/journal.pone.0298411)
Supplement: S4 Table — (DOCX) [file pone.0298411.s010.docx]

**Table S4. Detailed mean and range calculations for all injury categories and for all validity and reliability outcomes**

|  | | **Injury Type** | | | | | | | | |  | | |
| --- | --- | --- | --- | --- | --- | --- | --- | --- | --- | --- | --- | --- | --- |
|  |  | Self-harm injuries | Abuse | Transport and Pedestrian Injuries | Poisoning | Hand and wrist injuries | Brain injuries | Spinal cord injuries | Lower extremities injuries | Multiple (total body) injury types reported | **Mean Values Across Injury Types** | **Range of Values Across Injury Types** |  |
| **Validity Outcomes** | Sensitivity | 44.8% + 40% + 18.7% + 38.5% / 4  = **35.50%** | 31.8% + 72.6% + 62.5% / 3  = **55.63%** | (96.7% + 90.2% + 98.3% + 98.3% + 95.1%) / 5 = 95.72%  (25.0% + 35.6% + 45.0% + 30.0% + 30.0%) / 5 = 33.12%  (87% + 98%) / 2 = 92.5%  (95.72% + 33.12% + 92.5%) / 3  = **73.78%** | (81.2% + 89.7% + 91.5% + 94.9%) / 4 = 89.33%  (83% + 76%) / 2 = 79.5%  (89.33% + 79.5%) / 2  = **84.41%** | **96.0%** | 72.9% + 81% + 6.8% / 3 =  **53.57%** | 89.8% + 80% + 14.3% + 22.9% + 10.5% + 15.2% + 0.9% + 6.7% + 5.7% + 33.3% + 75% + 69% + 50% / 13  = **36.41** | (93% + 2.7% + 95% + 93% + 1.3% + 94%) / 6 = 63.17%  (94% + 95%) / 2 = 94.5%  (71% + 38% + 0% + 33% + 50% + 40% + 20% + 63.17% + 87% + 94% + 94.5%) / 11  = **53.70%** | 88.1% + 66.3% + 69.8% + 89.5% + 5% + 76.1% / 6  = **65.80%** | (35.50 + 55.63 + 73.78 + 84.41 + 96.00 + 53.57 + 36.41 + 53.70 + 65.80) / 9  = **61.64%** | **35.5% - 96.0%** |  |
|  | Specificity | (96.6% + 98.2% + 94% + 92.6%) / 4 = **95.35%** | 84.6% + 90.8% + 87.3% + 90.8% + 87.3% / 5  = **88.16%** | (100% + 100% + 100% + 100% +  100%) / 5 = **100%** | (98.8% + 84% + 81.5% + 79.0%) / 4 = **85.83%** | **-** | **88%** | 25% + 6.7% + 98.8% + 100% + 99.8% + 99.8% + 99.5% + 100% + 99.8% + 99.1% + 97% + 98% + 98% / 13  **= 86.27** | 94% + 95% + 76% + 97% + 96% + 98% + 78%  **= 90.57** | **98.50%** | (95.35% + 88.16% + 100.00% + 85.83% + 88.00% + 86.27% + 90.57% + 98.50%) / 8  **= 91.58%** | **85.83% - 100%** |  |
|  | PPV | (89.8% + 91.9% + 97.3% + 97.9% + 98.4% + 98.9% + 97.9% + 98.8% + 98.9%) / 9 = 96.64%  66.8% + 83.6% + 56.3% + 29.1% + 96.64% + 88.9% + 38.2% + 51.2% / 8  **= 63.84%** | (97% + 93% + 86% + 94% + 91% + 81% + 89% + 86% + 72% + 59% + 52% + 24%) / 12 = 71.06%  71.06% + 76% / 2  **= 73.53%** | (95% + 98%) / 2 = **96.5%** | (97.9% + 80.2% + 78.1% + 76.6%) / 4 = 83.2%  (67% + 71%) / 2 = 69%  (83.2% + 84% + 81% + 69% + 60.3% + 60.2% + 32.8% + 55.9% + 52.4%) / 9  **= 64.31%** | **92.0%** | (37.0% + 70.1% + 26.8% + 60.3% + 40.8% + 71.5% + 22.7% + 73.7%) / 8 = 50.36%  60.6% + 74% + 93% + 50.36% + 90% + 100% + 100% + 33.3% + 71.4% / 9  **= 74.74%** | 96.3% + 30% + 76.2% + 100% + 91.7% + 94.1% + 33.3% + 100% + 85.7% + 89.7% + 97% + 93% + 76% / 13  **= 81.77%** | (68% + 6.2% + 53% + 80% + 10% + 72%) / 6 = 48.2%  (100% + 91%) / 2 = 95.5%  (50% + 91% + 0% + 75% + 43% + 50% + 5% + 48.2% + 87% + 100% + 95.5%) / 11  **= 58.61%** | 34.3% + 95.5% + 93.8% + 93.3% + 5.5% + 92.6% / 6  **= 69.17%** | (63.84% + 73.53% + 96.50% + 64.31% + 92.00% + 74.74% + 81.77% + 58.61% + 69.17%) / 9  **=74.94%** | **58.61% - 96.5%** |  |
|  | NPV | 91.9% + 87.7% + 73.7% + 95% / 4  **= 87.08%** | **44.6%** | - | 92.08% (87.9% + 91.9% + 93% + 95.5%) / 4 **= 92.08%** | - | **92.8%** | 10% + 40% + 75% + 86% + 93% / 5  **= 60.80%** | 98% + 54% + 99% + 87% + 97% + 97% + 95% / 7  **= 89.57%** | **94.4%** | (87.08% + 44.60% + 90.83% + 92.80% + 60.80% + 89.57% + 94.40%) / 7  **= 80.01%** | **44.6% - 94.4%** |  |
| **Mean Values Across Validity Outcomes** | | (35.50% + 95.35% + 63.84% + 87.08%) / 4  **= 70.44%** | (55.63% + 88.16% + 73.53% + 44.60%) / 4  **= 65.48%** | (73.78% + 100% + 96.50% + 0%) / 4  **= 67.57%** | (84.41% + 85.83% + 64.31% + 90.83%) /4  **= 81.34%** | (96.0% + 0% + 92.0% + 0%) / 4  **= 47.00%** | (53.57% + 88.0% + 74.74% + 92.8%) / 4  **= 77.28%** | (36.41% + 86.27% + 81.77% + 60.80%) / 4  **= 66.31%** | (53.70% + 90.57% + 58.61% + 89.57%) / 4  **= 73.11%** | 65.80% + 98.50% + 69.17% + 94.40% / 4  **= 81.97%** |  | |  |
| **Range of Values Across Validity Outcomes** | | **35.50% - 95.35%** | **44.60% - 88.16%** | **0% - 100%** | **64.31% - 90.83%** | **0% - 96.0%** | **53.57% - 92.8%** | **36.41% - 86.27%** | **53.70% - 90.57%** | **65.80% - 98.50%** |  |  |  |
| **Reliability Outcomes** | Krippendorff’s alpha | **-** | **-** | **-** | **-** | **-** | **-** | **-** | (0.593 + 0.313) / 2 = 0.453  **0.453** | **-** | **= 0.453** | - |  |
|  | Cohen’s kappa | 0.481 + 0.478 / 2  **= 0.480** | **0.818** | (0.93 + 0.88) / 2 = 0.905  (0.90 + 0.97) / 2 = 0.935  (0.91 + 0.98) / 2 = 0.945  0.905 + 0.935 + 0.945 / 3  **= 0.928** | (0.74 + 0.73) / 2 = 0.735  **0.735** | - | - | 0.7 + 0.68 + 0.56 / 3  **= 0.647** | (0.485 + 0.604 + 0.294 + 0.255) / 4 = 0.41  (0.97 + 0.93) / 2 = 0.95  0.26 + 0.41 + 0.95 / 3  **= 0.540** | 0.75 + 0.77 + 0.15 / 3  **= 0.557** | (0.480 + 0.818 + 0.928 + 0.735 + 0.647 + 0.540 + 0.557) / 7  **= 0.672** | **0.480 – 0.928** |  |
|  | Fleiss’ kappa | **-** | **0.63** | **-** | **-** | **-** | **-** | **-** | **-** | **-** | **= 0.63** | - |  |
| **Mean Values Across Reliability Outcomes** | | **0.480** | 0.818 + 0.63 / 2  **= 0.724** | **0.928** | **0.735** | - | - | **0.647** | 0.453 + 0.540 / 2  **= 0.497** | **0.557** |  | |  |
| **Range of Values Across Reliability Outcomes** | | - | **0.63 – 0.818** | - | - | - | - | - | **0.453 – 0.540** | - |  |  |  |
