# Supplementary material for: Validity and reliability International Classification of Diseases-10 codes for all forms of injury: A systematic review
Source: PLoS One. 2024 Feb 29;19(2):e0298411. doi: 10.1371/journal.pone.0298411 (PMC10903801; doi:10.1371/journal.pone.0298411)
Supplement: Attachment — Submitted filename: PLOS_ONE_ICD_10_Validity_Reliability_Review.docx [file pone.0298411.s012.docx]

**PLOS ONE- RE:** Validity and reliability International Classification of Diseases-10 codes for all forms of injury: a systematic review

Thank you for the opportunity to review this paper:

The aim of this systematic review was to investigate the validity and reliability of ICD-10 codes for external-cause injuries.

This is important to gain a broader knowledge of the overall accuracy of these codes in identifying the correct diagnoses and also of the reproducibility among different coding individuals. The study showed that the validity and reliability of ICD-10 external cause injury codes vary based on the injury types coded and the outcomes examined, and overall, they only performed moderately well. It also highlights the need for future studies in order to increase the performance of the ICD codes (AI, etc.).

I congratulate the authors for the present important work, particularly because it’s the first overall analysis (systematic review) of the validity and reliability of ICD-10 codes for external-cause injuries.
The methodology, presentation and interpretation of the data presented is adequate and relevant. I only have a few comments to clarify:

**Introduction:**

Nothing to add.

**Methods:**

- Personally, I would prefer to remove the definitions of sensitivity, specificity, PPV and NPV (lines 170-180). It makes it hard to read and is somehow obvious.
- - In/Exclusion criteria: Consider listing the criteria for either exclusion or inclusion, but not in both sections, as this is redundant for the reader (peer-reviewed, publications in English, full text available)

**Results:**

- Line 243-249: Consider to rephrase this section. For the reader it’s confusing that you mention that a total of 33 articles were assessed for eligibility, whereby six were excluded. After the explanation why you excluded those 6 articles you mention another three articles that were excluded from the original search. However, finally you state that 27 articles were included for this systematic review of external cause of injury codes (33-6-3=24).

**Discussion:**

- Good structure of discussion, nothing to add.

**Figures and Tables**

- Provide them in sufficient quality. The diagrams provided for review were blurry.
