# Supplementary material for: Validity and reliability International Classification of Diseases-10 codes for all forms of injury: A systematic review
Source: PLoS One. 2024 Feb 29;19(2):e0298411. doi: 10.1371/journal.pone.0298411 (PMC10903801; doi:10.1371/journal.pone.0298411)
Supplement: Attachment — Submitted filename: Response to Reviewers ICD-10 Systematic Review Jan 15.24.docx [file pone.0298411.s013.docx]

**Dr. Michael D. Cusimano**	injuryprevention@smh.ca

**DEPARTMENT OF SURGERY	St. Michael’s Hospital**

**DIVISION OF NEUROSURGERY	38 Shuter St., Bond Site,	2nd Flr, Toronto, Ontario	Canada M5B 1A6**

January 15, 2024

FAO: Dr. Chenette and Dr. Simmen

Editor of PLOS One

RE: Manuscript titled “Validity and reliability International Classification of Diseases-10 codes for all forms of injury: a systematic review”

Dear Dr. Chenette and Dr. Simmen,

We would like to thank you for allowing us the chance to amend and re-submit our article for potential publication in your reputable journal *PLOS One*. We have undertaken the suggested changes and introduced several revisions. We amended the limitations section, the future directions, and clarified the PICOS framework and the inclusion/exclusion criteria. We generated the figures with clearer quality and attached these documents. Additionally, we addressed the questions asked by the reviewers.

Please find below the point-by-point reply to the reviewers.

Please let me know if you have any other comments or suggestions.

Sincerely,

**Michael D. Cusimano MD, PhD**

Professor of Neurosurgery Education and Public Health

St. Michael’s Hospital, University of Toronto

Toronto, ON, Canada

**Reviewer Comments:**

Reviewer #1: PLOS ONE- RE: Validity and reliability International Classification of Diseases-10 codes for all forms of injury: a systematic review

Methods:
- Personally, I would recommend to remove the definitions of sensitivity, specificity, PPV and NPV (lines 170-180). It makes it hard to read and is somehow obvious.

We thank the reviewer for their comments and for the review of our study. We have removed these definitions.

- - In/exclusion criteria: Consider listing the criteria for either exclusion or inclusion, but not in both sections, as this is redundant for the reader (peer-reviewed, publications in English, full text available,etc.)

We have removed the exclusion criteria description and kept only the inclusion criteria paragraph.

Results:
- Line 243-249: Consider to rephrase this section. For the reader it’s confusing that you mention that a total of 33 articles were assessed for eligibility, whereby six were excluded. After the explanation why you excluded those 6 articles you mention another three articles that were excluded from the original search. However, finally you state that 27 articles were included for this systematic review of external cause of injury codes (33-6-3=24).

We have amended this section for clarity, which is outlined on page 13, lines 258-266.

Figures and Tables
- Provide them in sufficient quality. The diagrams provided for review were blurry.

We have provided the diagrams with improved quality for better readability.

We thank the reviewer for their review of our study.

Reviewer #2: Thank you for the opportunity to review this manuscript submitted to PLOS ONE.

1. Page 4, line 71: The abbreviation “UI” should be written out in full.

We thank the reviewer for their comments and for the review of our study. We have amended this accordingly.

2. Page 6, line 35: “All studies included must have been peer reviewed, primary articles, published in English, examining humans, and have full-text available.” As stated by the authors, ICD-10 codes are used worldwide. The inclusion of artciles in English only may have led to a selectio bias. This should be mentioned in the limitations.

We have added this to the limitations section which can be found on page 33, line 675-676.

3. Page 11, line 11: In my understanding, PICOS would rather be structured as follows: Population: injured patients, intervention: ICD-10 coding, comparator: physician diagnosis/chart review, outcome: validity (measured as sensitivity, specificity, PPV, and NPV) and reliablity (Krippendorff's alpha, Cohen’s Kappa, and/or Fleiss’ kappa). Please explain the structure of the PICOS framework in more detail.

We have re-structured the PICOS framework as suggested and explained this in more detail, so this section is clearer (page 11, lines 184-188).

4. Page 13, line 222: “Ranges and mean values were calculated and reported for each of the outcomes in all the injury categories to provide an overall estimate of the validity and/or reliability of the ICD-10 codes for those injuries.” Usually the standard deviation is given for the mean and the range or interquartile range for the median. What was the rationale to report the mean with a range?

We reported the mean with a range rather than the standard deviation since as mentioned, multiple injury outcomes only had one study which examined that specific injury outcome. Thus, standard deviation was not applicable to these outcomes. For the other injury outcomes, in our opinion, with a small number of outcomes included in the calculations (all <13 outcomes for each injury category), standard deviation would not add to these outcomes. However, the range was reported to provide more information on the range of outcomes included in the mean calculations to show the scope of values included. This is outlined in the summary figures for each outcome where the ranges were reported with error bars depicting the range of outcomes included in the mean calculations (if multiple outcomes were reported).

5. Results, Table 3: As it seems, some outcomes were only reported in one study. (e.g., sensitivity for poisoning, specificity for brain injuries, PPV for hand and wrist injuries, and NPV for abuse). I would suggest mentioning this as an additional limitation in the discussion section.

We have amended the limitations accordingly and included this, which can be found on pages 33 and 34, lines 680-686.

6. The discussion section starts with the key study strengths. As a reader, I would be more interested in the key findings of the study at the beginning of the discussion. The authors may want to consider discussing the main findings of this review at the beginning of the discussion.

We have amended the discussion by discussing the main findings at the beginning of this section and moving the key study strengths after the discussion on page 33, lines 666-671.

7. This review investigated the validity and reliablity of ICD-10 codes for external cause injuries. It would also be interesting to investigate the validity and reliability of ICD-10 codes for specific injuries by body region. I understand that this is outside the scope of this review. However, the topic could be addressed in future studies.

We agree that this would be interesting to investigate, and we have added this to the future directions section on page 32, lines 638-640.

We thank the reviewer for their review of our study.
